# Rapid Stress Relief of Ti-6Al-4V Titanium Alloy by Electropulsing Treatment

**DOI:** 10.3390/ma18245555

**Published:** 2025-12-11

**Authors:** Aprilia Aprilia, Jin Lee Tan, Zixuan Ling, Vincent Gill, Paul Williams, Martyn A. Jones, Wei Zhou

**Affiliations:** 1 Rolls-Royce@NTU Corporate Laboratory, Nanyang Technological University, 65 Nanyang Drive, Singapore 637460, Singapore; 2 School of Mechanical and Aerospace Engineering, Nanyang Technological University, 50 Nanyang Avenue, Singapore 639798, Singapore; 3 Rolls-Royce Singapore Pte. Ltd., 1 Seletar Aerospace Crescent, Singapore 797565, Singapore; 4 Rolls-Royce PLC, P.O. Box 31, Derby DE24 8BJ, UK

**Keywords:** electropulsing treatment, rapid stress relief, residual stress, Ti-6Al-4V

## Abstract

This study investigates the effectiveness and underlying mechanisms of electropulsing treatment (EPT) for rapid stress relief of Ti-6Al-4V titanium alloy. Stress relief is an essential step in manufacturing processes to ensure long component lifespan. Residual stress accumulation within a component is often undesirable, as it may lead to premature failures. Currently, the stress relief of titanium alloys is typically carried out using an annealing heat-treatment process in a vacuum furnace. However, this method is time-consuming, usually requiring several hours. In this paper, an alternative fast stress relief method by EPT was investigated. A controllable pulsing treatment using alternating high density pulsing current with short pulse width was carried out. Results showed that EPT is effective in relieving residual stress in Ti-6Al-4V alloy. Up to 90% of the surface residual stresses induced by shot peening were successfully relieved by EPT with a treatment duration of only 114 ms. Reductions of low-angle grain boundaries (2–10°), local misorientation, and deformed grains were observed, while no significant grain growth or phase transformation was found. The stress-relief mechanism of EPT is attributed to the combined effects of dislocation movement driven by electron wind force (EWF), dislocation creep at elevated temperatures, and dislocation glide due to local yielding of residual stress under high-temperature conditions. The temperature rise during EPT was identified as a significant factor enabling stress relaxation.

## 1. Introduction

Titanium alloys are commonly used in aerospace applications because of their desirable properties, such as a high strength-to-weight ratio and excellent corrosion resistance [1]. During both the manufacturing and repair of aerospace titanium components, residual stresses are often introduced due to plastic deformation and thermal gradients generated by these processes [2]. Residual stress, especially tensile residual stress, is undesirable, as it can contribute to premature component failure [3]. Therefore, a stress-relief process is typically required to ensure adequate component lifespan.

Stress relief of titanium alloys is typically carried out through an annealing heat treatment in a vacuum furnace. However, this process is time-consuming and often requires several hours. Eshawish et al. [4] performed stress-relief heat treatment on selective laser melting-fabricated Ti-6Al-4V samples at 704 ± 14 °C for 2 ± 0.25 h in vacuum. Similarly, Emanuelli et al. [5] conducted stress-relief heat treatment inside a vacuum furnace for their laser powder bed fusion Ti-6Al-4V samples, applying a treatment temperature of 600 °C for 4 h. Kovalchuk et al. [6] also carried out stress-relief annealing of Ti-6Al-4V samples produced by wire-electron beam melting, using a treatment temperature of 650 °C for 4 h in a vacuum. Xie et al. [7] evaluated the effectiveness of stress relief in welded Ti-6Al-4V samples heat-treated at two temperatures (500 °C and 650 °C) for various soaking durations (0, 10, 30, 60, and 120 min). They found that the degree of stress relief increased with both temperature and soaking time. Under their optimal heat-treatment condition, 650 °C for 120 min, the residual stress was reduced from 550 MPa (as-welded condition) to 50 MPa. The required soaking duration for furnace-based stress relief is highly dependent on sample thickness [8]. According to AMS2801C, stress-relief furnace heat treatment for titanium alloy components should be performed at 593 °C with a soaking duration of 2 h ± 15 min [9].

In this study, a rapid stress-relief method using a controllable electropulsing treatment (EPT) is proposed. Unlike conventional heat treatment, where heating results from the transfer of thermal energy from the exterior to the interior of the material, heating in EPT is generated internally through resistive Joule heating induced by the applied current. Due to this difference in heating mechanism, EPT can achieve uniform through-thickness heating with a significantly higher heating rate, depending on the input current.

Several studies have demonstrated the use of EPT for residual stress relief. Pan et al. [10] observed a reduction in residual stress in ISO 5832-7 high-elastic cobalt-based alloy after high-density electropulsing treatment using a capacitor discharge pulse waveform with a peak current of 650 A and a pulse duration of 500 µs. The stresses were reduced from 485 MPa to 271 MPa. Similarly, Haque et al. [11] reported reductions in residual stress in a welded joint of 316L stainless steel using low-frequency direct-current (DC) electropulsing; compressive stresses were reduced from −493 MPa to −308 MPa. Xiang et al. [12] also demonstrated stress relief in quenched X80 steel samples treated with electropulsing at various current densities (3942–5562 A/mm^2^). They found that stress relief increases with increasing current density, with approximately 80% reduction achieved at 5562 A/mm^2^. They concluded that electropulsing accelerates dislocation annihilation by drift electrons by influencing the thermally activated dislocation motion in addition to exerting electron wind forces to the dislocation. Gudur et al. [13] applied in situ electropulsing during wire arc additive manufacturing to reduce residual stresses in the built component. DC pulses with a peak current of 800 A were applied to each deposited layer, resulting in a 24.0–29.4% reduction in residual stress compared with untreated samples. A study from our research group by Bhowmik et al. [14] also showed that EPT can promote dislocation disentanglement in the cold-worked layer of shot-peened AISI 1020 steel.

Some studies have explored the use of EPT on Ti-6Al-4V; however, these efforts focused on enhancing the alloy’s plasticity and formability. Shivaprasad et al. [15] reported a reduction in V-bending force in Ti-6Al-4V subjected to pulsed direct-current electropulsing. Subrahmanyam et al. [16] investigated the effect of electric pulse parameters on the draw-bending formability of Ti-6Al-4V and found that increasing current density reduced the required forming force even at constant energy density. Li et al. [17] showed improvements in bending formability and tensile elongation with electropulsing, successfully eliminating cracking during bending. Similarly, Ao et al. [18] reported enhanced tensile elongation after electropulsing, attributing it to recrystallization of Ti-6Al-4V. They suggested that electropulsing accelerates phase transformation and recrystallization at relatively low temperatures compared with conventional heat treatment.

To date, no study has investigated the use of EPT for stress relief of Ti-6Al-4V titanium alloy. This represents a significant gap, as effective rapid stress-relief techniques would be highly valuable for aerospace manufacturing and repair applications. In this work, the effectiveness and mechanisms of EPT for rapid stress relief in Ti-6Al-4V alloy are investigated. The effect of Joule heating-induced temperature rise during EPT on the residual stress relaxation is examined. EPT was conducted using a bespoke system capable of supplying a controllable current waveform to the material. Various alternating-current cycles of high current density and short pulse duration were tested. For the pre-treatment stressed coupon, shot-peened coupons were utilized. Although the compressive residual stresses generated by shot peening are typically beneficial, this method was selected because it produces consistent and uniform surface residual stresses in the samples. This would make it easier to study the stress relief effectiveness of different EPT parameters and to evaluate the microstructure changes before and after EPT. However, it is to be noted that the intended application of EPT is not to relieve stresses induced by shot peening.

## 2. Materials and Methods

### 2.1. Shot-Peened Ti-6Al-4V Plate

Annealed Ti-6Al-4V plates with dimensions of 100 mm × 20 mm × 3.5 mm were shot-peened. The annealed Ti-6Al-4V sample has the typical annealed Ti-6Al-4V microstructure, consisting of equiaxed α grains with intergranular β phases. Shot peening was performed using cast steel shots ASR 230 at an Almen intensity of 0.097 mmA with 100% coverage. ASR 230 refers to aerospace regular-hardness shots (45–52 HRC) with a nominal size of 0.023 inches, corresponding to a diameter of approximately 0.6–0.7 mm.

**Figure 1:**
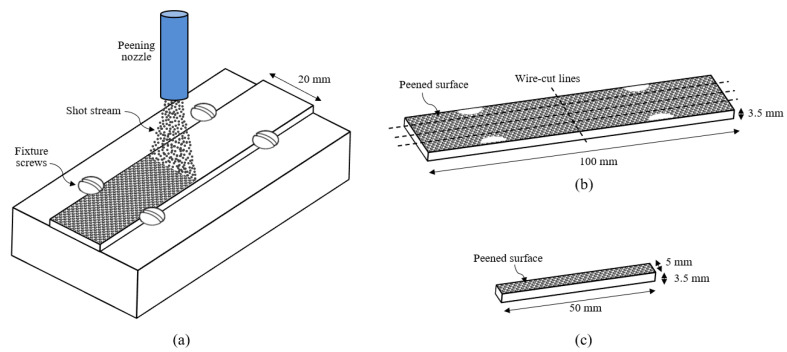
(**a**) Shot peening setup illustration, (**b**) wire-cutting dimensions of shot-peened samples, and (**c**) EPT samples’ dimensions.

Figure 1 illustrates the shot peening setup and wire-cutting dimensions. During peening, the annealed Ti-6Al-4V plate was secured in a fixture using four screws. After peening, the plate was cut into smaller coupons (50 mm × 5 mm × 3.5 mm) for electropulsing treatment (see Figure 1c). Residual stress measurements were conducted before and after EPT.

### 2.2. Residual Stress Measurement

**Figure 2:**
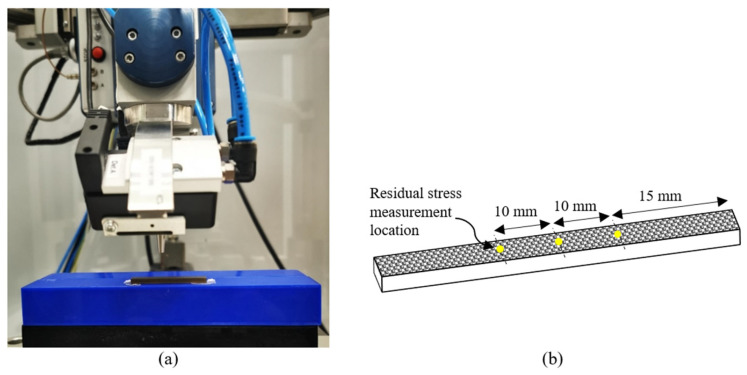
Residual stress measurement: (**a**) measurement setup and (**b**) measurement locations on the samples.

A Stresstech G3R X-ray diffractometer was used to measure the residual stress. The modified Chi mode method, as described in ASTM E2860-20 [19], was employed, together with a Cu-Kα X-ray source and a 2 mm collimator. Measurements were conducted at a tube voltage of 30 kV and a tube current of 10 mA, with an exposure time of 100 s. The diffraction angle (2θ) was measured at 140° for the (213) crystallographic plane. Prior to measurement, the equipment was calibrated using a stress-free powder and a standard stress reference specimen. Residual stress was measured at three different locations for each sample condition. Figure 2 shows the residual stress measurement setup and the measurement locations on the samples.

### 2.3. Electropulsing Treatment (EPT)

**Figure 3:**
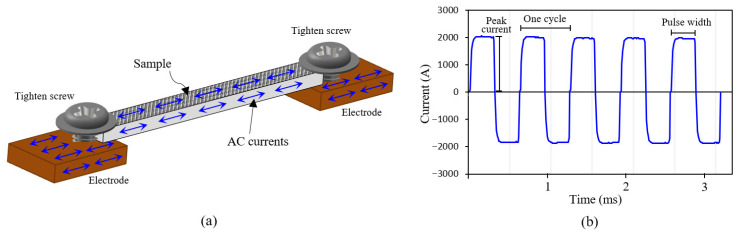
Electropulsing treatment: (**a**) clamping setup and (**b**) square alternating current waveform.

EPT was performed using a bespoke system capable of generating a controllable alternating-current (AC) square waveform. AC current was selected because the periodic reversal of electron flow is expected to enhance stress-relief effectiveness. Figure 3 shows the sample clamping setup and the alternating-current waveform used. The sample was placed on top of two copper electrodes and secured with two screws (Figure 3a), with the shot-peened surface facing upward. The sample was mechanically constrained by the screws throughout the treatment. During a localized or non-localized electropulsing treatment, the treated region is always constrained, either by the surrounding material or, as in this study, by the electrode clamping points. A square AC waveform was then supplied at a constant peak current for a specific number of cycles (Figure 3b). Each cycle consists of one positive and one negative current pulse. The duration of peak current in each pulse is referred to as the pulse width.

**Figure 4:**
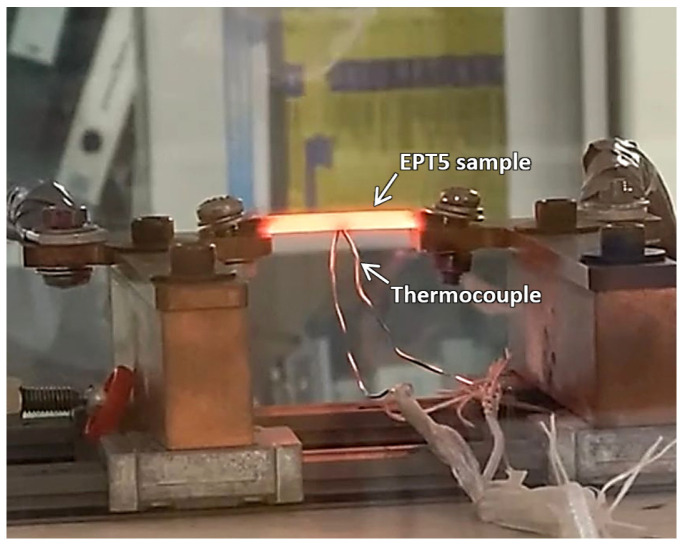
Photograph of the EPT5 sample during electropulsing.

Five electropulsed samples were used for the investigation. The detailed parameters are shown in Table 1. The peak current and pulse width were fixed at 2000 A and 3 ms, respectively. These high current and short pulse width parameters were chosen to maximize electron–material interaction. The number of cycles varied from 10 to 31 cycles. An increase in cycles will increase the energy input and treatment duration, thereby resulting in higher sample temperatures. Figure 4 shows a photograph taken during the EPT of sample EPT5, in which the sample glowed bright red during pulsing. To record the peak temperature, a K-type thermocouple was attached to each sample during the EPT process. The peak temperatures recorded for all samples are presented in Table 1.

### 2.4. Microstructure Characterization

Microstructures of as-received and as-peened samples were examined using a JEOL JSM-7600F field emission scanning electron microscope (FESEM) (Tokyo, Japan). The samples were ground with sandpaper of up to 4000 grit and subsequently polished using a 1 μm diamond suspension followed by a mixture of OP-S Struers colloidal silica suspensions and 19% hydrogen peroxide. They were then etched with Kroll’s reagent (2–6% nitric acid, 1–3% hydrofluoric acid and 91–97% water) obtained from Best Chemical Co (S) Pte. Ltd. (Singapore, Singapore) for approximately 10 s.

For backscattered electron (BSE) imaging, the samples underwent the same preparation steps except for the etching process. Etching was omitted to avoid inaccuracies in β-phase size measurement that may arise from variations in etchant–material interactions across different samples. BSE micrographs were obtained at an accelerating voltage of 20 kV using the same FESEM system. β-phase size measurement was performed using the ImageJ 1.53 e (NIH, Bethesda, MD, USA).

For the electron backscattered diffraction (EBSD) analysis, EBSD maps were acquired using the same FESEM equipped with an Oxford Instruments detector (Abingdon, UK). A 20 kV accelerating voltage and a 0.1 μm step size were used. Grain boundary maps, kernel average misorientation (KAM) maps, and recrystallization fraction maps were generated.

## 3. Results

### 3.1. Residual Stress Results

Table 2 presents the residual stress values before and after electropulsing treatment, as measured using X-ray diffraction method described in Section 2.2. The residual stresses induced by shot peening were approximately −520 MPa (compressive) in both the longitudinal and transverse directions, and these values were consistent across all samples. After electropulsing, residual stress reduced in every sample. For sample EPT1, the residual stresses decreased by 23% (longitudinal) and 29% (transverse). In sample EPT2, the reductions were 53% (longitudinal) and 61% (transverse). Sample EPT3 exhibited reductions of 90% (longitudinal) and 91% (transverse). For samples EPT4 and EPT5, the residual stresses were almost completely relieved. These results show that increasing the number of electropulsing cycles enhances the degree of residual stress reduction. In this experiment, sample EPT4, treated with 23 cycles of 2000 A alternating current and a pulse width of 3 ms, achieved nearly complete relief of the initial compressive residual stress (−520 MPa), corresponding to more than a 97% reduction.

### 3.2. Microstructure Results

**Figure 5:**
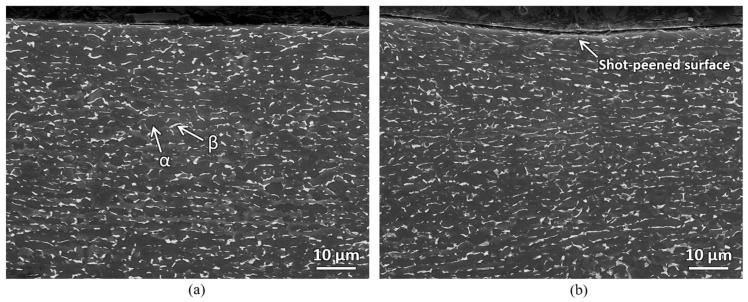
SEM micrographs of the sample: (**a**) before shot peening (**b**) after shot peening.

Figure 5 shows the SEM micrographs of the samples before and after shot peening (prior to EPT). As observed in the figure, the shot-peened sample exhibits a convex, curved surface. This curvature results from the repeated impact of the shots, which impart sufficient force to induce plastic deformation at the surface. The microstructure in the bulk region of the shot-peened sample remains similar to that of the as-received annealed plate, consisting of equiaxed α grains with intergranular β phases.

**Figure 6:**
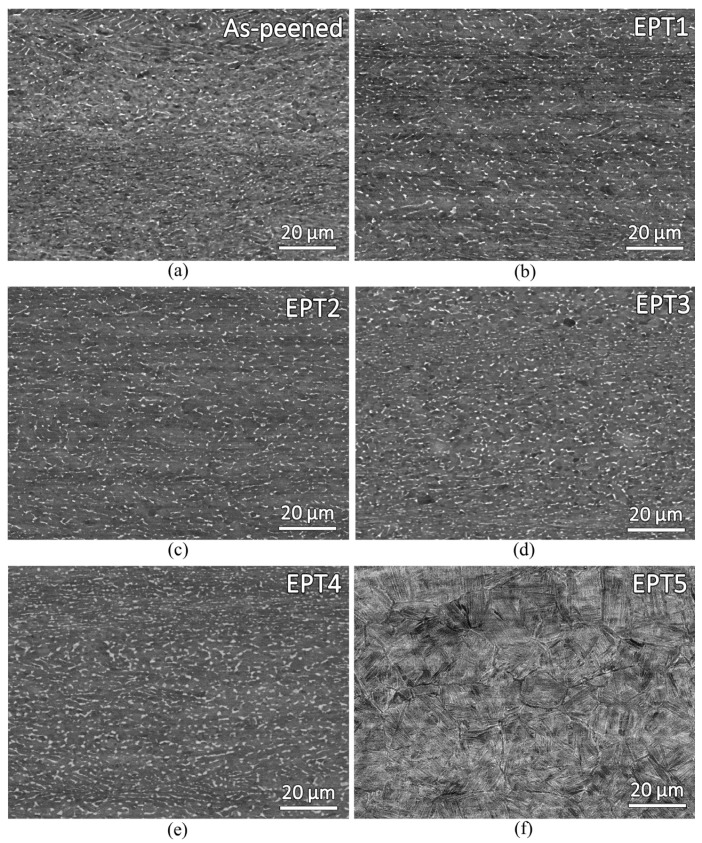
Backscattered electron (BSE) micrographs of the as-peened and electropulsing treated samples: (**a**) as-peened, (**b**) EPT1, (**c**) EPT2, (**d**) EPT3, (**e**) EPT4, and (**f**) EPT5.

In the EPT-treated samples, the curvature of the shot-peened surface remains visible; however, some microstructure changes are observed in the bulk region. Figure 6 presents the bulk microstructures of the as-peened sample and the EPT-treated samples (EPT1, EPT2, EPT3, EPT4, and EPT5). As shown, the microstructure of sample EPT5 has changed significantly after treatment, exhibiting lamellar α colonies within prior β grains. This type of microstructure is typically observed in Ti-6Al-4V alloy when it is heated above the β-transus temperature [20]. For the remaining samples, the microstructures consist of equiaxed α grains with intergranular β phases, indicting that these samples were heated below the β-transus temperature. However, among these samples, the β-phase size in EPT4 appears to have increased.

Table 3 summarizes the observed microstructures and the average β-phase size. The β-phase in sample EPT4 is approximately 30% larger than that of the as-peened sample, based on measurements obtained using ImageJ software. This indicates that although the EPT applied to sample EPT4 (23 pulses) did not heat the material above the β-transus temperature, it still raised the temperature sufficiently to cause noticeable microstructural changes, particularly β-phase growth. To avoid significant microstructure alteration, EPT of Ti-6Al-4V alloy using a 2000 A alternating current with a 3 ms pulse width should be limited to fewer than 23 cycles. In this study, sample EPT3 satisfies this requirement, exhibiting no noticeable microstructural changes while still achieving a high residual stress reduction of approximately 90%.

**Figure 7:**
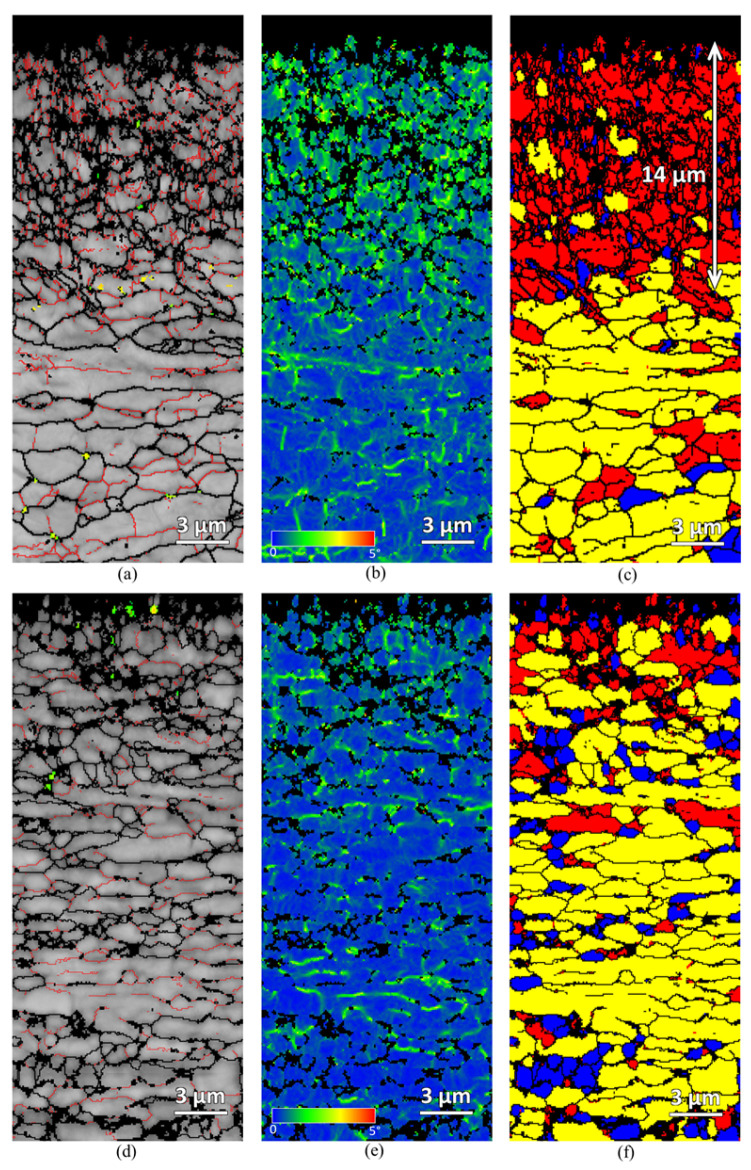
EBSD maps (grain boundary, KAM, and recrystallization fraction) of the as-peened sample (**a**–**c**) and sample EPT3 (**d**–**f**).

Further characterization of the shot-peened region was carried out using EBSD. Figure 7 presents the grain boundary, kernel average misorientation (KAM), and recrystallization fraction maps of the as-peened sample and sample EPT3 (~90% residual stress reduction). In the grain boundary maps, red contours represent the low-angle grain boundaries (LAGBs, 2–10°), while black contours indicate high-angle grain boundaries (HAGBs, >10°). In the KAM maps, green or yellow regions indicate high local misorientation, whereas blue regions indicate low local misorientation. In the recrystallisation fraction maps, red corresponds to deformed grains, yellow to substructured grains, and blue to fully recrystallized grains. As shown in Figure 7, the amount of LAGBs in the shot-peened region decreases after EPT. Likewise, both local misorientation and the number of deformed grains are reduced following treatment. In the recrystallization map of the as-peened sample (Figure 7c), the deformed layer produced by shot peening is approximately 14 μm. After EPT, the number of deformed grains within this region is significantly reduced.

## 4. Discussion

### 4.1. Electropulsing Treatment of Ti-6Al-4V Titanium Alloy

**Figure 8:**
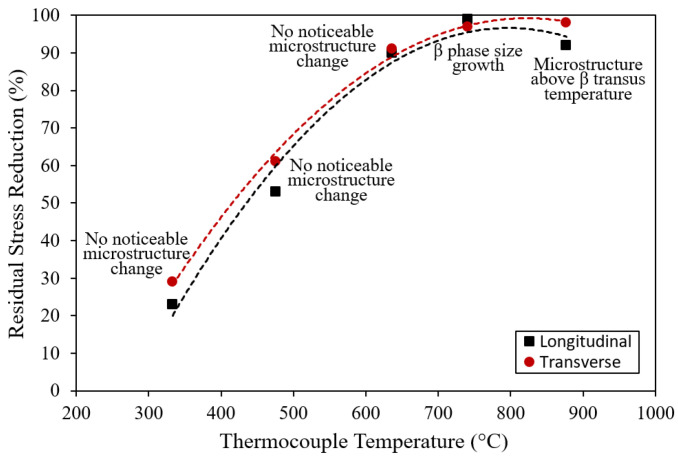
Summary of electropulsing treatment of Ti-6Al-4V alloy.

Figure 8 summarizes the EPT results for Ti-6Al-4V titanium alloy. Electropulsing treatment with a short duration of 114 ms was able to relieve approximately 90% of the compressive residual stress without causing any observable grain growth or phase transformation. In this case, the recorded peak temperature was 636 °C. For a longer treatment duration of 138 ms, corresponding to a recorded peak temperature of 741 °C, coarsening of the β-phase grains was observed. At an even longer duration of 186 ms, with a recorded peak temperature of 877 °C, a complete change in microstructure occurred, indicating that the material had been heated above the β-transus temperature. For Ti-6Al-4V, the β-transus temperature is typically reported to be between 970 and 1000 °C [21]. These findings indicate that the peak temperatures measured by the thermocouple are not accurate. This discrepancy is understandable, as the thermocouple used had a sampling rate of only 1 Hz. Due to the extremely rapid heating associated with electropulsing, the thermocouple was unable to capture the actual peak temperature experienced by the material. To estimate the true peak temperature, a numerical simulation was performed using the ANSYS Mechanical 2020 R2 (Ansys, Canonsburg, PA, USA) with an element mesh size of 0.25 mm.

Table 4 presents the peak temperatures measured by the thermocouple and those computed through numerical simulation, along with the corresponding treatment durations. As shown, the simulation-predicted peak temperature for sample EPT5 exceeds the β-transus temperature of Ti-6Al-4V (970–1000 °C), confirming that the simulated values more accurately reflect the true temperatures experienced during EPT. For sample EPT3, the ideal case with no observable grain growth or phase transformation, the simulation-computed peak temperature is approximately 785 °C.

### 4.2. Understanding Stress Relief Mechanisms of Electropulsing Treatment

Stress relief in a material occurs when dislocations move, thereby releasing stored strain energy and reducing internal stress. During electropulsing treatment, a high density of electric current passes through the material, resulting in a force acting on atoms and dislocations due to their interaction with the flowing electrons. This force is known as the electron wind force (EWF) [22]. The EWF functions as a mechanical force that pushes dislocation to move in the direction of electron flow. The movement typically occurs within individual grains, as movement across grain boundaries is often impeded by the dislocation networks present at these boundaries. During the process, dislocation accumulation, reorganization, and annihilation may take place at grain boundaries. The overall stress induced by EWF can be estimated using the equation given below [22,23].

(1) $$\sigma_{ed}=C_{ed}\cdot J,$$

where $\sigma_{ed}$ is the stress induced by EWF, $C_{ed}$ is the coefficient of electron–dislocation interaction, and J is the peak current density. In this work, the peak current density is 1.14 × 10^8^ A/m^2^, and $C_{ed}$ for Ti atoms is 3.2 × 10^−8^ MPa/(Am^−2^) [22]. Therefore, the calculated EWF-induced stress is 3.65 MPa.

For a dislocation to move, the applied stress must exceed its Peierls stress or, for large-scale dislocation motion, the critical resolved shear stress (CRSS) [24]. For Ti-6Al-4V titanium alloy, the CRSS at room temperature is estimated to be about 380 MPa for prismatic slip {1010}<1120> of a polycrystalline Ti-6Al-4V alloy [23]. However, Salem et al. [25] reported that the CRSS of Ti-6Al-4V single-colony samples decreases to about 40–80 MPa for the same prismatic slip at 815 °C. Similar to yield strength, CRSS decreases with increasing temperature. The calculated EWF-induced stress of 3.65 MPa from Equation (1) is significantly lower than the reported CRSS values, even at elevated temperatures. Therefore, additional mechanisms must contribute to dislocation motion and the resulting residual stress reduction during EPT.

**Figure 9:**
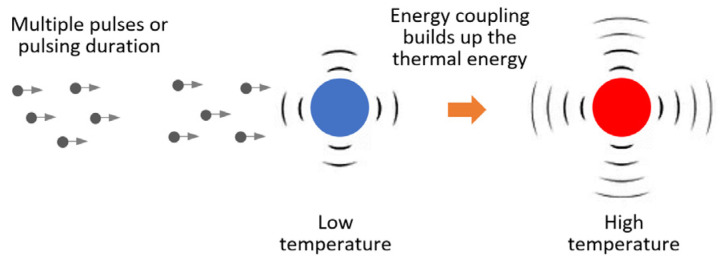
Joule heating in electropulsing treatment.

As shown in Table 2 and Table 3, increasing the number of pulsing cycles or the treatment duration enhances residual stress relief, even when the peak current density remains constant. This indicates that pulsing cycles and treatment duration also play roles in stress reduction. With a larger number of cycles of a longer pulsing duration, more thermal energy accumulates (energy coupling) in the material through Joule heating, which arises from the conversion of electrical energy into heat as current passes through a resistive material (Figure 9). As the number of cycles or treatment duration increases, the material temperature rises (Equation (2)), promoting increased dislocation motion. This behavior follows the principles of material heat capacity and Joule’s law of electrical heating.

(2) $$∆T=\frac{I^{2}Rt}{mc},$$

where ∆T is the increase of temperature, I is electric current, R is material resistance, t is pulsing duration, m is the mass of the material, and c is the specific heat capacity.

In addition to the overall temperature rise caused by Joule heating, there may also be a transient temperature increase during pulsing, an effect that occurs only under pulsed current conditions and not during steady direct-current heating. The transient temperature rise depends on the pulse width $t_{p}$ and the thermal diffusivity α of the material [26]. For the temperature range relevant to this study, the thermal diffusivity of Ti-6Al-4V varies from 2.7 mm^2^/s to 8.5 mm^2^/s [27]. With a pulse width of $t_{p}$= 3 ms, the characteristic diffusion length, $l_{diff}\approx \sqrt{\alpha t_{p}}$, is approximately 90–160 µm. This diffusion length is much smaller than the sample thickness (3.5 mm), indicating the likelihood of localized temperature rises within the material due to the thermal flux generated as the pulsed current ramps up and down. Furthermore, localized heating may also occur at dislocation cores due to ‘local’ Joule heating [28]. The high electrical resistivity at dislocation cores can lead to localized heat nucleation, arising from microscopic resistivity inhomogeneities. These three phenomena, global Joule heating, transient pulsing-induced heating, and local dislocation-core Joule heating, collectively contribute to significant temperature rises at the dislocation cores during electropulsing treatment.

In summary, stress relief during electropulsing treatment can be attributed to three possible mechanisms. First, dislocation movement may be induced by the EWF, which drives dislocations in the direction of electron flow. However, as discussed earlier, the magnitude of the EWF-induced stress in this study is insufficient to produce large-scale dislocation motion on its own. Second, stress relief may occur due to the increased vacancy diffusion at elevated temperatures which allows for dislocation climb (also referred to as dislocation creep) and therefore dislocation annihilation. This process is believed to be one of the primary mechanisms for conventional thermal (static) stress relief [29]. Unlike EWF-driven motion, vacancy diffusion and thermal stress relief are non-directional and time-dependent. The increase of temperature in the material during electropulsing can result from the ‘global’ Joule heating, transient temperature rise from pulsing, and the local temperature rise at dislocation cores. The third possible mechanism of the stress relief is dislocation glide by local yielding of residual stresses at high temperatures. As the material temperature increases, the CRSS and yield strength of Ti-6Al-4V decrease. When the reduced yield strength falls below the existing residual stresses, plastic deformation and dislocation glides occur which results in a fall of residual stresses to the level of the yield strength.

Without further evidence, it is likely that all three mechanisms and potentially other unidentified mechanisms are interacting to result in the stress relief recorded in this study. This study shows that in using electropulsing treatment for residual stress reduction, the increase of material temperature by increasing the pulsing cycle and treatment duration is an important factor for the residual stress reduction. Unless substantially higher current densities are applied, the influence of the electron wind force is far less significant than the temperature-driven effects. In practical applications, much higher current density is not feasible due to safety concerns, large component sizes, and the substantial electrical energy required. Although electropulsing treatment involves temperature rise similar to the conventional furnace heat treatment, EPT offers several advantages, including fast heating rates, significantly shorter exposure times of the material at elevated temperatures, and the capability for localized treatment. These characteristics highlight the potential of EPT as a viable and efficient method for residual stress relief.

## 5. Conclusions

In this study, rapid stress relief of Ti-6Al-4V titanium alloy parts using a controllable electropulsing treatment was investigated. Electropulsing treatments with varying numbers of square alternating-current cycles, using a peak current of 2000 A and a pulse width of 3 ms, were investigated. The conclusions drawn from this study are as follows:

(1) Electropulsing treatment of Ti-6Al-4V alloy for a short period of 114 ms (19 pulse cycles) can relieve approximately 90% of the compressive residual stress without resulting in any observable grain growth or phase transformation. Further optimization of treatment parameters such as current density and pulse width can be further explored to enhance the residual stress relief.
(2) EBSD analysis showed reductions in low-angle grain boundaries (2–10°), local misorientation, and deformed grains in the electropulsed samples.
(3) Stress relief during electropulsing treatment is attributed to a combination of three possible mechanisms: EWF-induced stress, dislocation creep, and dislocation glide by local yielding. The increase of material temperature in electropulsing treatment is an important aspect of the stress relief, as it enables dislocation creep and local yielding to occur.

## Figures and Tables

**Table 1:** EPT parameters of the five investigated samples along with the measured peak temperatures by thermocouple.

Sample	Peak Current (A)	Pulse Width (ms)	Number of Cycles	Thermocouple Measured Peak Temperature (°C)
EPT1	2000	3	10	333
EPT2	2000	3	13	475
EPT3	2000	3	19	636
EPT4	2000	3	23	741
EPT5	2000	3	31	877

**Table 2:** Residual stresses (RS) before and after EPT.

Sample	Pre-EPT RS (MPa)		Post-EPT RS (MPa)		RS Reduction ^a^	
	Longitudinal	Transverse	Longitudinal	Transverse	Longitudinal	Transverse
EPT1	−521 ± 17	−591 ± 26	−401 ± 8	−421 ± 7	23%	29%
EPT2	−506 ± 26	−530 ± 17	−238 ± 39	−205 ± 15	53%	61%
EPT3	−489 ± 1	−502 ± 2	−47 ± 8	−43 ± 5	90%	91%
EPT4	−513 ± 9	−523 ± 11	6 ± 7	−16 ± 6	99% *	97%
EPT5	−513 ± 6	−527 ± 30	40 ± 6	13 ± 4	92% *	98% *

Residual stress sign indication: positive value → tensile; negative value → compressive. ^a^ Percentage reductions are calculated using absolute value. * Residual stress has changed from compressive to tensile.

**Table 3:** Microstructures and average β-phase size of the samples.

Sample	Observed Microstructure	Average β-Phase Size (µm)
As-peened	Equiaxed α grains + intergranular β-phases	0.73
EPT1	Equiaxed α grains + intergranular β-phases	0.77
EPT2	Equiaxed α grains + intergranular β-phases	0.78
EPT3	Equiaxed α grains + intergranular β-phases	0.77
EPT4	Equiaxed α grains + intergranular β-phases	0.95
EPT5	Lamellar α colonies within prior β grains	-

**Table 4:** Treatment duration and peak temperatures of the samples (measured by thermocouples and computed by simulation).

Sample	Treatment Duration (ms)	Thermocouple-Measured Peak Temperature (°C)	Simulation-Computed Peak Temperature (°C)
EPT1	60	333	451
EPT2	78	475	571
EPT3	114	636	785
EPT4	138	741	969
EPT5	186	877	1270

## Data Availability

The original contributions presented in this study are included in the article. Further inquiries can be directed to the corresponding author.
